# The Book World of Medicine and Science

**Published:** 1905-12-23

**Authors:** 


### The Book World of Medicine and Science.

Sanatoria for Consumptives. By F. Rufenacht Walters, M.D., M.R.C.P. (Swan, Sonnenschein and Co., 1905. Pp. 389. Third Edition.)

The plan of this work is excellent and its scope wide; and the fact that three editions have been called for within six years shows that it has been appreciated. To those who were not fortunate enough to see one of the earlier editions we may say that in addition to an outline of the treatment of phthisis in the open air, the work contains chapters on diet, climate, soil, construction, decoration and furniture of sanatoria, and descriptions of a vast number of these hospitals both at home and abroad. Some of these descriptions are necessarily rather short. Illustrations of upwards of fifty sanatoria are given; but a great many of these are merely elevations or perspective drawings, and, although useful as a means of advertising sanatoria, they are nearly worthless to those who are studying the subject with a view to construction. When another edition is called for we hope these pretty pictures will be omitted, and ground plans of the buildings substituted. It is immaterial whether these ground plans are good or bad, as the architect should have his attention called to what he is to avoid as well as to what he may copy. At the outset Dr. Walters explains that sanatoria are not intended for the treatment of those who are seriously ill, and that such cases are in the first instance better treated in a hospital, nursing home, or in their own homes. We are not sure that we fully agree with Dr. Walters in this statement, but it may point to the advisableness of having a separate section or annexe for the treatment of bad cases.

In these days sanatoria for consumptives are springing up in many directions. The good results obtained therein are no longer doubtful, and it is only the lack of funds which has prevented the building of many more sanatoria and the enlargement of existing ones. The work is therefore opportune. It has been often shown that the old ideas held by physicians that consumptives needed a warm climate as an essential to successful treatment were erroneous; and the essentials as regards the site of the buildings are clearly given by Dr. Walters. They are (1) pure and bracing air, (2) shelter from cold winds, (3) a climate which is sufficiently fine to make outdoor life attractive, and (4) a warm and well-drained soil. Beyond doubt these conditions may be obtained in many parts of our own islands, and it is once more emphasised by Dr. Walters that it is fresh air and medical supervision that are needed rather than a fine climate. The value of Alpine resorts is admittedly good in many cases, but we think the author is right when he says that a certain reactive power should be present in all patients sent to these high latitudes. The advantages and disadvantages attending sanatoria composed of small chalets and those consisting of one building are fairly well placed before the reader. We are convinced that for the large majority of cases the chalet is preferable, because the drawbacks do not refer to the patient, but to the administration, and can be overcome. A table is given showing the results in 5,000 cases treated at the Brehmer Sanatorium; and the interesting and hopeful fact is shown that when treatment is begun in the first stage of the disease 27 per cent. are cured and 31 per cent. nearly cured. In the second stage 6 per cent. were cured and 14 per cent. were nearly cured. The great difficulty which the physician has to fight against is the too early removal of the patient from the sanatorium. If this could be banished the cures would be more numerous.

Nodal Fever. By Alfred Austin Leudon, M.D. (London: Baillière, Tindall, and Cox. 5s. net.)

The author applies the term "nodal fever" to the condition generally known as erythema nodosum, and this little treatise is devoted to proving that erythema nodosum is in reality an acute specific infectious fever. Dr. Leudon brings forward clinical evidence to show that the course of the disease is marked by a prodromal stage of fever and general malaise, usually lasting about a fortnight: then comes the stage of eruption, marked by the appearance of the erythema nodosum on the limbs, followed by a period of convalescence. The disease "also occurs under conditions that strongly suggest infection from one case to the next." The proofs for this infectiousness are not convincing; since no precautions are usually taken to isolate cases, the disease ought to be much commoner than it is. It seems as though there must be, in addition to this hypothetical infectiousness, which we must presume to be slight, a tendency, a diathesis, rheumatic or otherwise, which must be present in an individual before the disease can be contracted: this would account for several cases of the disorder occurring in the same family, since each of the members of the family may be assumed to have inherited this tendency or diathesis. The book is well worth perusal.

